# LiDAR Point Cloud Recognition of Overhead Catenary System with Deep Learning

**DOI:** 10.3390/s20082212

**Published:** 2020-04-14

**Authors:** Shuai Lin, Cheng Xu, Lipei Chen, Siqi Li, Xiaohan Tu

**Affiliations:** College of Computer Science and Electronic Engineering, Hunan University, Changsha 410082, China

**Keywords:** catenary inspection, deep learning, LiDAR, OCS, point cloud recognition

## Abstract

High-speed railways have been one of the most popular means of transportation all over the world. As an important part of the high-speed railway power supply system, the overhead catenary system (OCS) directly influences the stable operation of the railway, so regular inspection and maintenance are essential. Now manual inspection is too inefficient and high-cost to fit the requirements for high-speed railway operation, and automatic inspection becomes a trend. The 3D information in the point cloud is useful for geometric parameter measurement in the catenary inspection. Thus it is significant to recognize the components of OCS from the point cloud data collected by the inspection equipment, which promotes the automation of parameter measurement. In this paper, we present a novel method based on deep learning to recognize point clouds of OCS components. The method identifies the context of each single frame point cloud by a convolutional neural network (CNN) and combines some single frame data based on classification results, then inputs them into a segmentation network to identify OCS components. To verify the method, we build a point cloud dataset of OCS components that contains eight categories. The experimental results demonstrate that the proposed method can detect OCS components with high accuracy. Our work can be applied to the real OCS components detection and has great practical significance for OCS automatic inspection.

## 1. Introduction

Since the 21st century, the high-speed railway has gradually become an important transportation mode. As a faster and safer travel option, the high-speed railway system faces huge challenges in operation and maintenance. To ensure safety and reliability, the periodical inspection and maintenance of the railway system are essential [1]. With the increase of operation length, the cost of maintenance has become a huge burden. Therefore, the efficiency and reliability of inspection are vital.

**Figure 1:**
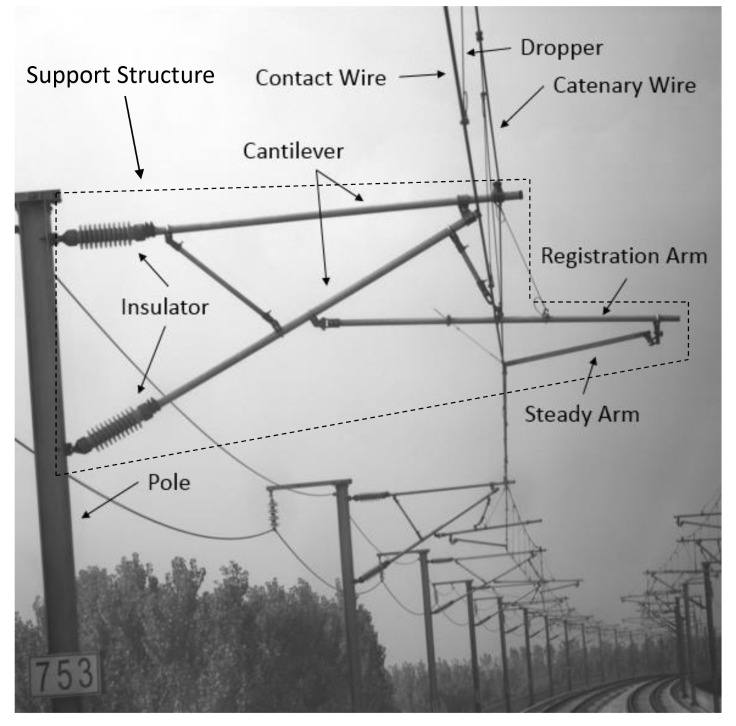
OCS structure.

The overhead catenary system (OCS) undertakes the task of providing stable power for the high-speed train. It consists of the catenary wire, dropper, contact wire, pole, and support structure, as shown in Figure 1. The support structure supports and fixes the catenary wire. The catenary wire and dropper are used to hang the contact wire to keep it level, and the pantograph above the train contacts with the contact wire to get electricity. The geometric parameters of the components directly affect the quality of the current collection and driving safety. The pantograph may hit the steady arm if the slope of the steady arm is too small [2], and improper height of the contact wire is bad for current collection and increases the wear between the pantograph and the contact wire. Due to long-term operation in the outdoor environment and the impact of the pantograph, the components might become loose and damaged, and geometric parameters also change, which would affect the normal operation of OCS. The OCS inspection aims to find the abnormal conditions of components, provide the basis for maintenance, and ensure safe operation. Geometric parameters measurement and defect detection are important tasks of OCS inspection. Whether the geometric parameters meet the requirement and the states of components determine if the catenary system works normally.

Traditionally, manual operation is the most common OCS inspection method. The staff walk along the rail track and work with portable measuring devices [3]. This inspection method is quite costly, inefficient, prone to error, and requires high technical workers. It is hard to meet the large maintenance requirements. To improve efficiency and decrease maintenance costs, many researchers have developed some automatic inspection equipment and intelligent detection methods. The mobile non-contact detection method is one; a camera or LiDAR (Light Detection And Ranging) is mounted on mobile equipment to collect the information of OCS, then automatically identify particular OCS components in the data, measure specific parameters or detect their fault states. In recent years, deep learning has been widely used in computer vision [4] because of its excellent performance, and many scholars have adopted this method to process the 2D images of OCS to improve the detection efficiency and accuracy [5,6,7]. However, there are few works based on the point cloud. The image-based detection method is often used to detect the abnormal state of components, such as slack, breakage, and missing. Compared with 2D-image, the point cloud contains three-dimensional coordinate information of the scene, which is useful to measure geometric parameters [8,9]. Therefore, the study of automatically processing the catenary point cloud has great significance.

Automated analysis of catenary point clouds mainly includes automatic recognition of OCS components from the point clouds and parameter measurement based on the recognition results and spatial information, and the first is necessary and most important. The OCS consists of many components, and the railway environment is very complicated, which makes identification quite difficult. The deep learning can extract high-dimension features automatically and efficiently, and a large number of models based on it are proposed for the point cloud recognition task [10]. Those models have superior performance and reach a higher benchmark. So the deep learning is promising for point cloud classification and recognition of OCS and can be adopted to realize automated inspection.

The objective of this work is to identify the catenary components with point clouds based on deep learning. The contributions are as follows:

1. Introduce the deep learning to the multiple catenary components recognition based on the point cloud, such as catenary wire, dropper, contact wire, pole, and support structure. The support structure is further subdivided into the insulator, cantilever, registration arm, and steady arm.
2. Propose a recognition framework to deal with the problems about training and recognition caused by large-scale point cloud in the catenary scene.
3. Design a segmentation model to improve the recognition accuracy of some OCS components in complex scenarios.

The experimental results show the effectiveness and performance of the method, providing a way for target identification in automated OCS inspection.

This paper is organized as follows. Section 2 discusses related work about catenary recognition and point cloud. Section 3 introduces the details of our method. Section 4 shows and discusses the experimental results. The conclusions and future work are presented in Section 5.

## 2. Related Work

The point cloud recognition algorithms mainly include edge-based, region growing, model fitting, clustering-based, and machine learning [11]. Now, many studies about point cloud recognition of catenary are based on these methods. The work [12] proposed the region growing algorithm for segmenting the railway scene, then adopted the KD tree and the closest point searching algorithm to achieve the classification of the segmented point cloud. In [13], the catenary support structure was divided by R-RANSAC (Region-Random Sample Consensus) algorithm and Euclidean clustering, the geometric parameters of the steady arm could be calculated with the space vector information of segmented linear part. The cantilever is a crucial part of the catenary support equipment. For monitoring the state of the cantilever structure, the authors in [14] adopted the improved LCCP (locally convex connected patches) to segment the cantilever structure and presented a new RANSAC (random sample consensus) algorithm to measure the geometry parameters of cantilever structure. The proposed approach could satisfy the practical inspection requirement. To automate the processing of catenary point clouds obtained with the MMS (mobile mapping system), Pastucha [15] utilized RANSAC to detect and classify the cantilevers, support structures, and catenary wires, then improved the classification result with a modified DBSCAN clustering algorithm. The above methods are based on strict handcrafted features from geometric rules and constrained by designed prior knowledge. The extracted features have a limited ability to describe the statistical relation of data, so these methods are mainly for specified detection targets. The methods based on machine learning can model the representative feature distribution from the training data and identify multiple targets. Aiming at improving the recognition result of complex railway scenes, Jung et al. [16] designed a classifier based on the MrCRF (multi-range Conditional Random Field) and SVM (support vector machine) to identify ten key components, introducing neighbor information for the misclassification caused by similar features. The MrCRF only takes spatial information within limited ranges and not considers the shape and size variance, so Chen et al. [17] introduced the hierarchical CRF model to solve the problems. They also utilized the fully connected CRF model to gather all contextual information. Both methods realize the simultaneous recognition for multiple catenary components based on the point cloud, but not further divide the support structure.

Deep learning can extract features automatically from the data. It promotes the development of computer vision greatly and has been applied to object classification, object localization, semantic segmentation. Many researchers have used this method to process the captured 2D-images of OCS for efficiently locating the components and identifying the faults. In [18], authors applied deep learning algorithms to locate catenary support components in the images and detect their faults, finding that deep learning methods can identify multiple categories and are greatly superior to traditional methods based on artificial features in accuracy and timeliness. Chen et al. [19] built a cascaded CNN architecture to detect the tiny fasteners in catenary support devices from the high-resolution images and reported their missing states, with a good detection accuracy and robustness. Due to the long-term impact of the environment, defects in the components are inevitable. Aiming at achieving automated insulator defect identification, Kang et al. [20] presented a detection system based on a deep convolutional neural network, which has an excellent detection performance and can be applied to the inspection system. There are many other studies based on deep learning processing the catenary image, but few for the point cloud.

The point cloud is irregular and unordered, so it is hard to utilize deep learning for point cloud recognition. Inspired by feature learning approaches based on deep learning used for image recognition, researchers proposed some methods to process point cloud. These methods [21,22,23,24] transform the point clouds to voxel-based representation or 2D images as input to deep neural networks, obtaining better recognition results compared with traditional methods based on handcrafted features. However, these methods generally miss much spatial information when transforming and take a long time to train the model. To improve computing efficiency and keep spatial information, some deep learning models based on raw point cloud are proposed. PointNet [25] is a pioneering network architecture that works on raw point cloud and has been used for 3D object recognition [26,27,28]. It improves the performance of point cloud classification and segmentation. However, it does not take the local structure information within neighboring points into account, which leads to loss of relevant information and a bad segmentation result in large scenes. Drawing inspiration from PointNet, many researchers study how to improve the semantic segmentation result by constructing local relationships among points. Point cloud recognition research is driven by the development of deep learning techniques, which provides a theoretical basis for point cloud recognition based on deep learning in intelligent OCS inspection.

## 3. Methodology

### 3.1. Target Components

The OCS structure is shown in Figure 1. There are eight important components: catenary wire, dropper, contact wire, insulator, pole, cantilever, registration arm, and steady arm, respectively. Their reliability is vital to the long-term stable operation of OCS, so they are the main targets of inspection. The OCS is so large-distributed that it is very difficult to get access to each of its components. Therefore, the inspection systems are mainly based on vehicle-mounted optical sensors, which allows measurement without physical contact, and improves detection efficiency and security. LiDAR is one of the optical sensors often used to collect spatial information of catenary for geometry inspection. To achieve automatic detection, automatic processing of massive amounts of data acquired by LiDAR is essential. Thus, our main goal was to recognize those key components in the point clouds.

### 3.2. Construction of Dataset

Dataset is indispensable to deep learning for the recognition tasks, providing a mass of labeled samples for verifying the proposed methods. Efficient and rich datasets can make the model robust and guarantee the results for subsequent processing. Some famous large-scale datasets such as S3DIS [29], ShapeNet [30], and ScanNet [31] are built for promoting the study of point cloud recognition, but there are no open point cloud datasets for the catenary system. To verify the proposed method, we need to build a point cloud dataset.

**Figure 2:**
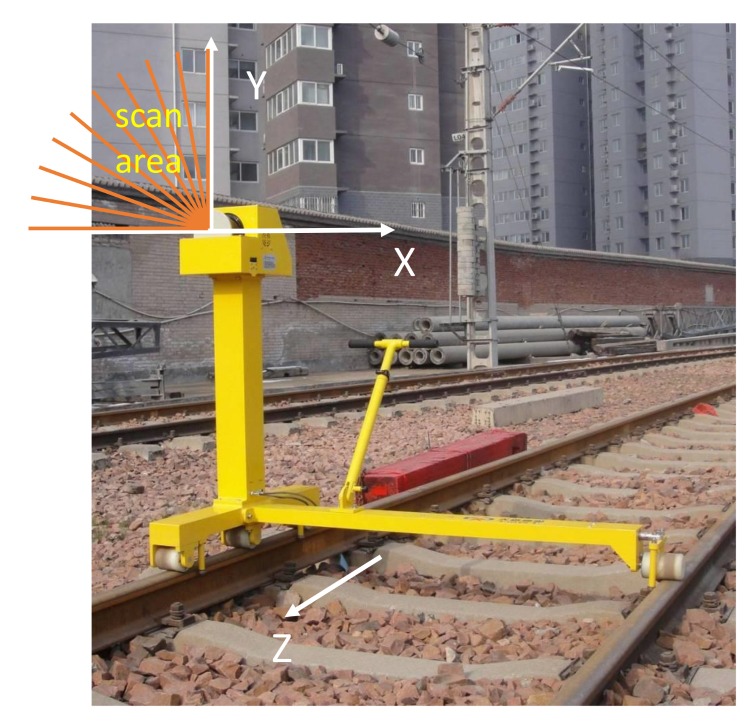
Data collection equipment and coordinate system.

**Figure 3:**
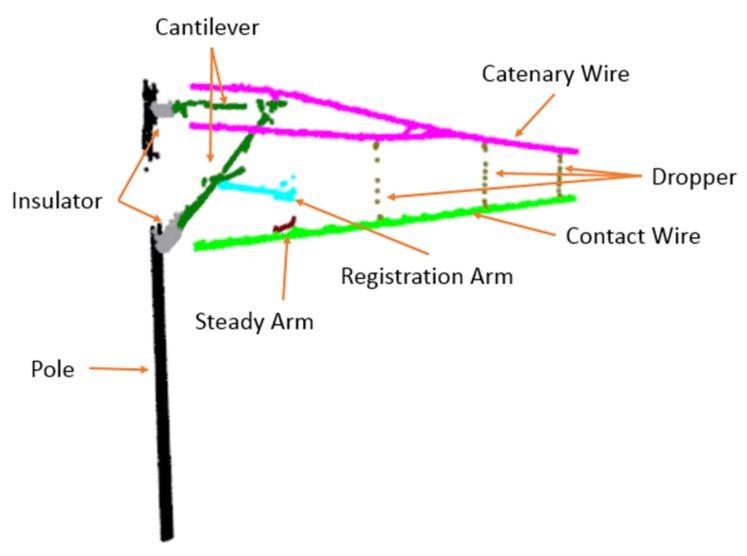
Annotated point cloud of catenary.

Figure 2 shows the mobile inspection equipment used to collect data and the coordinate system of the point cloud data. Portability, low-cost, automation, and good stability make the equipment widely used in OCS inspection. A SICK LiDAR is mounted on a device to gather catenary data and its scanning direction towards the sky. The range of scanning angle is $90^{\circ }$ to $180^{\circ }$, and the scanning frequency is 25 Hz. The x, y are calculated by the distance measured by the LiDAR and angle, the z is related to the movement distance of the equipment. The equipment is driven by a motor, and the max move speed is set to 1 m/s to collect intensive data. About 16 km point cloud data are filtered and labeled manually by self-made software. The annotated point clouds are visualized in Figure 3, and the interested components mentioned above are set to different colors.

### 3.3. Recognition Framework

The point clouds of the catenary are large-scale, and a few hundred meters of railway scene can contain more than 100,000 points. Therefore it is quite hard to train a good segmentation model with whole scene data, which requires high memory and training time costs. Besides, the amount of data for different types of components are very uneven, some are sparse like the dropper while some are dense like the pole, and training a model with the whole scene leads to a bad recognition result for sparse categories. For this problem, the most straightforward approach is to divide the whole scene into many groups, and each group contains multi frame point clouds, then use a segmentation model to recognize every group. If the numbers of frames in subdivided groups are same, it is hard to determine which number is better for recognition, because the number of frames for the pole is quite different from that for the dropper, and the data of a component are probably divided into two groups, which harms recognition accuracy due to the lack of data integrity.

**Figure 4:**
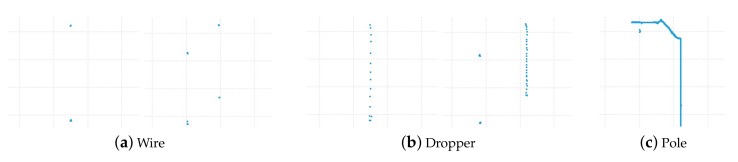
Context of the projection of a single frame point clouds onto the XY plane.

**Figure 5:**
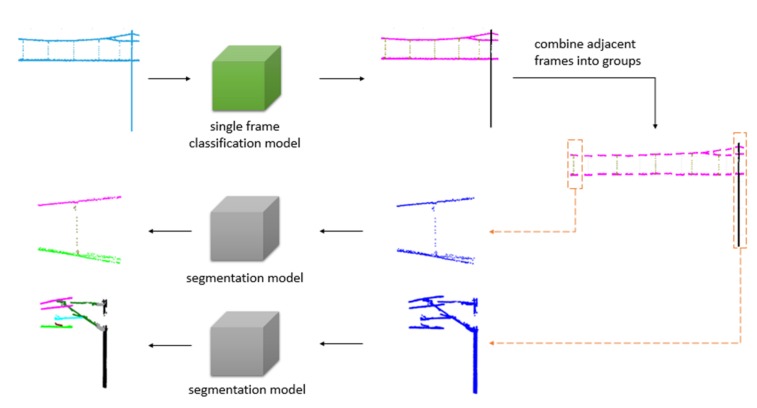
Recognition framework.

We propose a solution to address the problems mentioned above. The point cloud data of the whole scene is composed of many single frames. According to the scan range of the LiDAR, a single frame consists of point cloud data in the area of the XY plane from $90^{\circ }$ to $180^{\circ }$ at a certain movement distance. It can be classified into wire, dropper, and pole, as shown in Figure 4. The wire context contains the catenary wire and contact wire, the dropper context includes the wire and dropper, and the pole context consists of the wire, insulator, pole, cantilever, registration arm, and steady arm. Therefore, we use a single frame classification model to classify every frame, then combine adjacent frames based on classification results. The whole scene can be separated into many groups with different numbers of frames, and the data integrity of each component is guaranteed. After that, a segmentation model is adopted for recognition of the components in every group. The proposed method is shown in Figure 5 and Algorithm 1, and it has three advantages:

1. The large-scale catenary scene can be well compartmentalized for training and recognition. The segmentation model training costs and the difficulty of identifying components are reduced.
2. The subdivided groups can be identified by different segmentation models based on classification results. The simple model identifies the simple scenes (wire, simple dropper), while the complex model identifies the complex scenes (complex dropper, pole).
3. It is applicable for subsequent processing after data collection of the entire railway scene, and analysis during the inspection.

**Algorithm 1** Recognition Algorithm.1:initial S←Ø; //The set of single frames.2:initial C←wire; //The category of *S*.3:initial $M\leftarrow [M_{0},M_{1},M_{2}];$ //The minimum number of frames combined into a group.4:**repeat**5:    F=ReadSingleFrame(); //Read single frame data.6:    L=SingleFrameClassification(F); //Single frame classification model classifies the single frame.7:    FS,S,C=CombineSingleFrames(F,L,S,C,M); //Combine single frames into a group. The method is shown in Algorithm 2.8:    **if** FS≠null **then**9:        R=MultiFramesSegmentation(FS); //Segmentation model recognizes the components in the combined single frames.10:    **end if**11:**until** without single frame data.

### 3.4. Single Frame Classification Model

**Figure 6:**
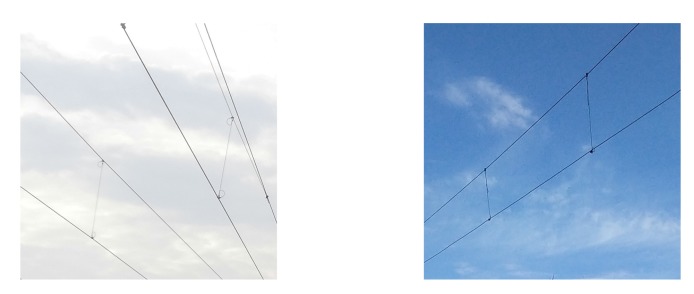
The wire scenes.

**Figure 7:**
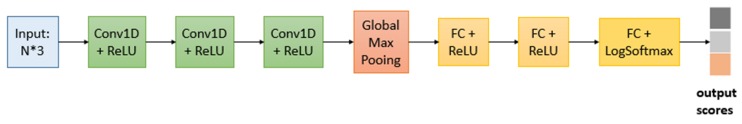
Single frame classification model.

According to the scanning direction and range of the LiDAR, the context of a single frame point clouds of catenary can be classified into wire, dropper, and pole, as shown in Figure 4. There are two sets of wires in some scenes, as shown in Figure 6, so there are two categories of wire and dropper context. Here, we use a convolutional neural network (CNN) as the single frame classification model to identify the context of each frame data. Its architecture was inspired by the PointNet [25] and shown in Figure 7.

The CNN takes the 3D coordinates of *n* points as input, applies feature transformations via three fully convolutional blocks, then combines the point features through global max pooling, finally outputs the classification scores of three classes with the three-layer perceptron. The one with the highest score is the predicted category. Each fully convolutional block contains a 1d-convolutional layer and a ReLU activation layer, and it is used as a feature extractor. The max-pooling is adopted to obtain the global features.

The CNN identifies the context of every single frame point cloud, and the adjacent frames are combined into groups based on the classification results. The combined method is shown in Algorithm 2. The set *S* is used to store the single frames. The minimum numbers of frames forming a group (wire, dropper and pole) are denoted by *$M_{0}$, $M_{1}$, $M_{2}$*. Every frame is added to the *S*. When the length of *S* is greater than or equal to the minimum numbers, the single frame point clouds in *S* form a group. For the wire, when $M_{0}$ consecutive frames are classified into wire, the $M_{0}$ frames are combined into a group representing the wire. When a single frame point cloud is classified into the dropper and pole, subsequent single frames are added to the *S* until the length of *S* meets the quantity required and the last single frame is classified into the wire. The reason for the last single frame is wire is to prevent the data of a component is divided into two groups. The combined result will be recognized by a point cloud segmentation model.
**Algorithm 2** Combine single frames into a group. **Input:**  (1) *F*: A single frame point clouds.  (2) *L*: The classification result, the value is wire, dropper or pole.  (3) *S*: The set of single frames. This output is used as the next input.  (4) *C*: The category of *S*. The value is wire, dropper or pole, and the default is wire. This output is used as the next input.  (5) *M*: The map consisting of *C* and the minimum number of frames forming a group representing *C*. **Output:**   result: The combined single frames. It may be null.
1:initial result←null;2:push the single frame into the set S.append(F);3:**if**L≠wire**then**4:    
C←L;5:**end if**6:**if**Length(S)≥M[C]**and**L=wire**then**7:    
result←S;8:    
S.clear();
9:    
C←wire;10:**end if**11:**return**result, *S*, *C*


### 3.5. Point Cloud Segmentation Model

**Figure 8:**
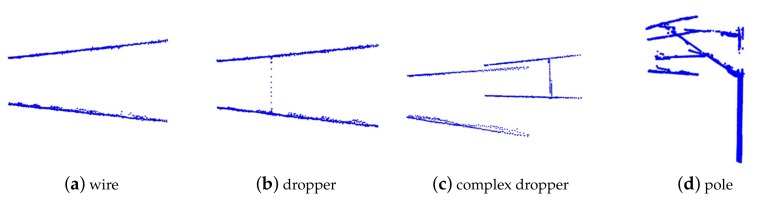
Categories of multi frame point clouds.

The single frames of point clouds are identified by the single frame classification model, and the adjacent frames are combined into a group based on their categories. The combined result can be classified into wire, dropper, and pole, as shown in Figure 8. There are eight types of components, and the segmentation model is used to recognize them in every group.

To work on point cloud directly and be invariant to input permutation, the PointNet extracts features for each point independently with the multi-layer perceptron (MLP), and it does not capture the relationship between adjacent points, which leads to limited recognition performance in the complex scenes. In the catenary system, the point clouds for some components are sparse, such as dropper and steady arm, lacking local neighborhood information causes a bad recognition result of them. Therefore it is necessary to combine the local neighborhood information to improve segmentation results in catenary scenes.

Many researches have proven that exploiting local structure information is vital for the success of convolutional architectures. The CNN captures local features progressively in a hierarchical way to get global features, which has better generalizability to complex cases. The data format of an image likes a regular grid, which makes the convolutional model can capture local features well and efficiently. However, point clouds are inherently irregular. Any permutation order of them does not change the spatial distribution and the represented shape. Thus, the methods used for extracting the local features need to maintain permutation invariance, and the results do not change with the order of point clouds.

Consider a *D* dimensional point cloud with n points, the feature of each point is denoted by $F_{x_{i}}$∈$R^{D}$, *i* = {1,…,n}. In the simplest case of *D* = 3, the feature of a point is represented by its 3D coordinates. In a deep neural network architecture, dimension *D* represents the feature dimension of a network layer, which is also the dimension of high-level features constructed directly or indirectly by the low-level features of the points.

For each point $x_{i}$, *i* = {1,…,n}, we take it as the center of a local region and construct its local neighborhood $N(x_{i})$ using the k-nearest neighbor (k-NN) algorithm, and extract the local feature with its neighbors $x_{j}$∈$N(x_{i})$, *j* = {1,…,k}. The local feature of point $x_{i}$ is denoted as $L_{x_{i}}$∈$R^{D}$, *i* = {1,…,n}. Based on the characteristics of point cloud mentioned above, the local feature should be invariant to the order of points, so the methods of combining features need to satisfy the permutation invariance. The common methods include summation, maximization, and so on, here we choose the summation. In the three-dimensional space, the geometric relation reflects the spatial information and is favorable to recognition, so we take the geometric relations between the center and its neighbors into account to extract the local feature more effectively. As a consequence, the local feature is formulated as

(1) $$L_{x_{i}}=\sum_{x_{j}\in N\left(x_{i}\right)}W_{ij}\cdot F_{x_{j}}$$

where $W_{ij}$ denotes the high-level relation between the center $x_{i}$ and neighbor $x_{j}$, and it is constructed by the geometric relation in the 3D space.

In the Euclidean distance algorithm, the correlation between points is related to their distance. The point $x_{j}$ is considered to the same class as the point $x_{i}$ when their distance is less than a threshold. The smaller the distance, the more likely the same class is, on the contrary, the possibility of falling in the same category is lower. Inspired by this, we use the distance to construct $W_{ij}$, and this process is described as

(2) $$W_{ij}=h_{\Theta }\left(d\left(x_{i},x_{j}\right)\right)$$

where $d(x_{i},x_{j})$ is the difference of the 3D coordinates, and $h_{\Theta }$ is a nonlinear function with learnable parameters Θ. Here we adopt a 2d-convolutional block to implement $h_{\Theta }$ due to its powerful ability for abstracting relation expression. The $d(x_{i},x_{j})$ is only relevant to both $x_{i}$ and $x_{j}$, $h_{\Theta }$ is shared to all neighbors, so $W_{ij}$ is independent to the irregularity of points, and the local feature $L_{x_{i}}$ is permutation-invariant.

**Figure 9:**
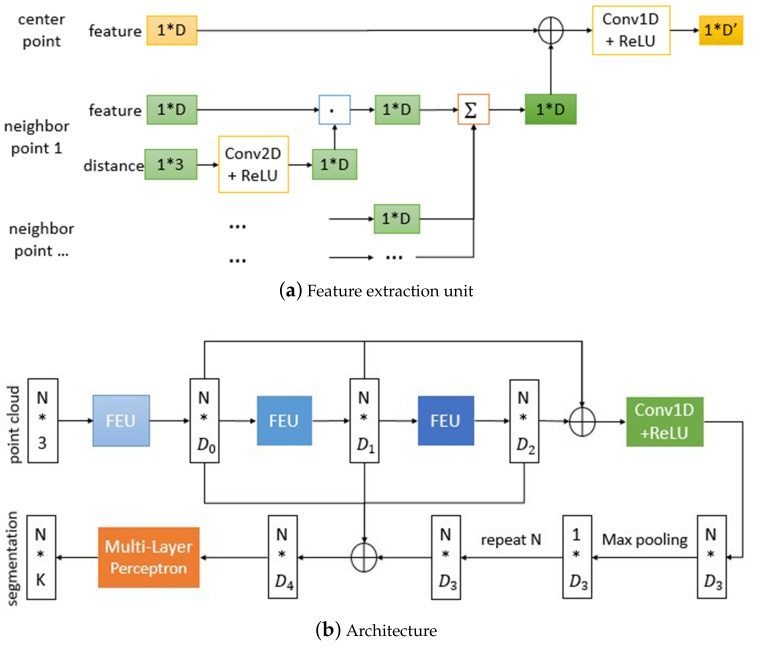
Segmentation model.

Finally the features of center and the local features are combined together to build high-level features $F_{x_{i}}^{{}^{\prime }}$∈$R^{D^{{}^{\prime }}}$ of center, as shown below:

(3) $$F_{x_{i}}^{{}^{\prime }}=g_{\Phi }\left(F_{x_{i}},L_{x_{i}}\right)$$

where $g_{\Phi }$ is a nonlinear function implemented by a 1d-convolutional block and similar to $h_{\Theta }$. The method mentioned above for extracting features of points is visualized in Figure 9a, it contains the features of center and neighbors, and takes their geometric relations into account. We take this method as the feature extraction unit (FEU) of our point cloud segmentation model, which is inspired by the network architecture for segmentation proposed in [25] and shown as Figure 9b. The model adopts the feature extraction unit to obtain the high-level features of points, then aggregates multilevel features to obtain the global features, after that concatenates the global and multilevel features as the input of the multi-layer perceptron, finally outputs per point scores for classifying each point.

## 4. Experimental Results and Analysis

In this section, we quantify the performance of the proposed method on the point cloud dataset of catenary mentioned in Section 3.2. The experimental environment is as follows: Deep learning framework Pytorch, Intel(R) Xeon(R) E5-2623 v4 Processor, 32 GB RAM, and a GPU NVIDIA Quadro P5000.

### 4.1. Single Frame Classification Performance

The proposed single frame classification model CNN has three fully convolutional blocks and three-layer perceptron. From the first to third convolutional blocks, the output channel is set to 64, 128, 1024, and the output channels of three-layer perceptron respectively are 256, 128, 3. About 6500 frames of the point cloud are selected in the experiment, 5200 samples are training set, and 1300 samples are testing set. During the training process, we use Adam optimizer with an initial learning rate of 0.001, and the learning rate decays by a factor of 0.5 every 25 epochs. The number of points per frame is different, so the batch size is set to 1. This paper uses the overall accuracy (OA) as the performance metric of the single frame classification model, and it is equal to the number of correctly classified frames (Correct Number) divided by the total number of frames (Total Number).

(4) $$Overall\;Accuracy=\frac{Correct\;Number}{Total\;Number}$$

**Figure 10:**
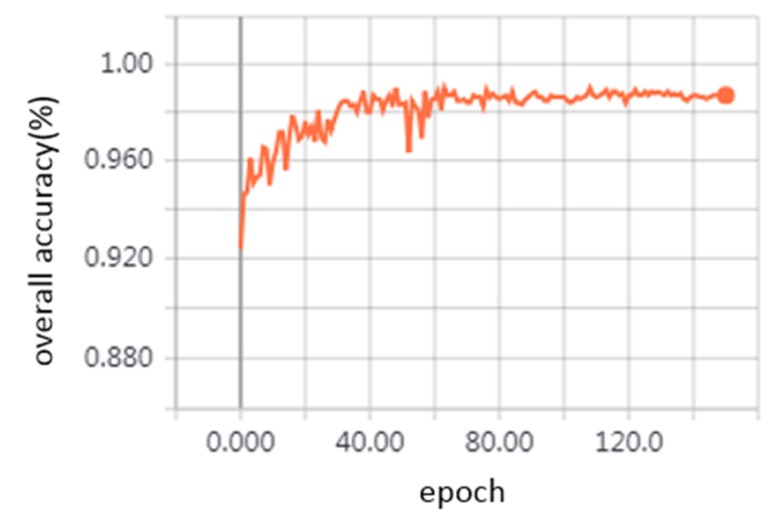
The overall accuracy of single frame classification model curves.

**Figure 11:**
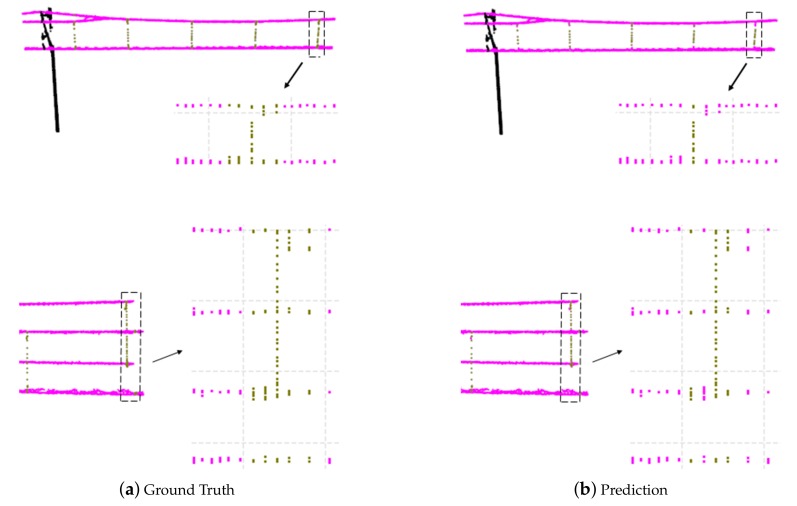
Classification results. The first column is the ground truth, and the second is prediction. The point clouds in a single frame are set to the same color based on the category of the single frame (wire in magenta, dropper in dark yellow, and pole in black). The arrows point to the projection of the point clouds near the dropper on the YZ plane (the coordinate system is the one of the collected data).

As shown in Figure 10, during the training process, the overall accuracy of the testing dataset increased rapidly and finally reached about 98.69%. Figure 11 visualizes the classification results for two examples. It can be observed that the single frames are well classified as wire, dropper, and pole. The points of the dropper in some single frames are so few that those frames are misidentified as the wire, but the single frames with many points of dropper can be classified correctly. The approximate positions of dropper are founded based on the frames with many points of the dropper, then a certain number of frames can be selected around them as the groups representing the dropper scenarios.

### 4.2. Segmentation Performance

The segmentation model has three feature extraction units, and the output channel of them is denoted by $D_{0}$, $D_{1}$, $D_{2}$. Here $D_{0}$, $D_{1}$, $D_{2}$ is set to 64, 64, 128. The output channel of the 1d-convolutional block is set to 1024. The multi-layer convolutional block consists of three 1d-convolutional blocks, and their output channel respectively is 256, 128, and 8. In the experiment, we select multi frame point clouds representing the wire, dropper, and pole from the dataset for training and verifying the segmentation model.

To evaluate the performance of our segmentation model, we compare it with PointNet. They are trained by Adam optimizer. The initial learning rate is 0.001, and it is divided by 2 per 50 epochs. The number of points in every multi frame data is different, so the batch size is set to 1. Due to uneven numbers of point clouds for catenary components, the overall accuracy is difficult to reflect the performance of models, so we calculate the per category and mean accuracy.

(5) $$Accuracy=\frac{Number\;of\;Correctly\;Classified\;Points}{Total\;Number\;of\;Points}$$

(6) $$Mean\;Accuracy=\frac{Summation\;of\;Accuracy}{Number\;of\;Categories}$$

In Table 1, we report per-category and mean accuracy. The *k* is the number of nearest neighbors. As we can see, when *k* is 16, the mean accuracy of our segmentation model is 97.01%, the recognition accuracy of each category is higher than with the use of PointNet, and the dropper and cantilever show a notably better accuracy. The catenary wire, contact wire, and pole obtain a good segmentation (about 99%) in both models, because of their simple structure and high density.

**Figure 12:**
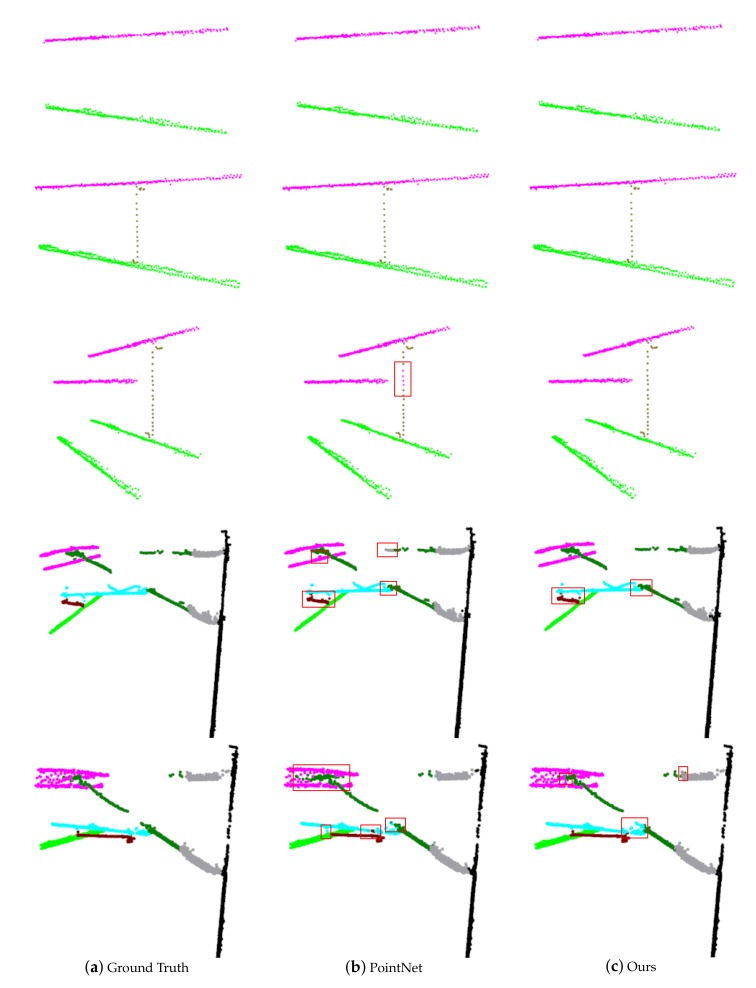
A comparison of the segmentation results. The first column is the ground truth. The second and third columns are the prediction of the PointNet and our model. The points belonging to different categories are colored differently (catenary wire in magenta, dropper in dark yellow, contact wire in green, insulator in gray, pole in black, cantilever in dark green, registration arm in cyan, and steady arm in dark red). The red areas are the main misclassified parts.

In Figure 12, we visually compare the results of PointNet and our model. The wire and simple dropper scenes are segmented well with both models. However, the PointNet classifies some points of the dropper as the catenary wire in the complex dropper scenes, because it only uses the individual point features and global features for recognition and does not capture the local neighborhood features. On the horizontal plane corresponding to the misidentified part, the points of the catenary wire are so dense that the extracted global features are mainly related to it, so the features combined by individual features and global features are mostly obtained from the catenary wire, which leads to the misidentification of the dropper. Due to the combination of local neighborhood information, our model achieves improved segmentation results.

### 4.3. The Segmentation Model Analysis

In this subsection, we analyze the effectiveness of the feature extraction unit and the different numbers *k* of nearest neighbors.

**Figure 13:**
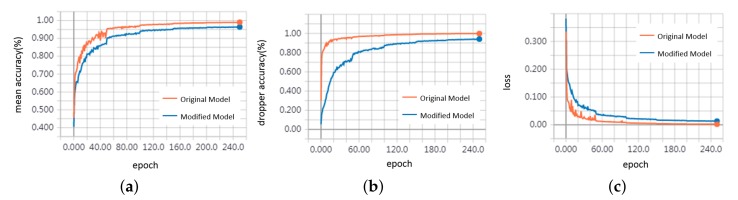
Training curves. (**a**) mean accuracy (**b**) dropper accuracy (**c**) loss.

To evaluate the performance of the feature extraction unit mentioned in Section 3.5, we replace it with a 1d-convolutional block, which processes each point independently and does not extract local neighborhood information. Table 1 shows the recognition accuracy comparison of the original and modified model. The accuracy and loss of the training process are shown in Figure 13. We can see that the model extracting local features outperforms the other one and has faster convergence speed. Due to sparsity, the dropper, registration arm, steady arm have lower accuracy when lacking neighborhood information. The local regions of points where the pole is connected to the cantilever or insulator contain some points belonging to the cantilever or insulator, and the extracted local features are quite related to them when the *k* is small, so the pole segmentation accuracy is reduced a little. Table 2 shows their detection time for each sample. The model extracting neighborhood information is slower because of the k-nearest neighbor (k-NN) algorithm for constructing the local neighborhood and the convolution operation of the local neighborhood.

To investigate the impact of the number *k* of nearest neighbors, we experiment with different *k*, as shown in Table 3. The segmentation model achieves 97.01% mean accuracy with *k* is 16. When *k* is decreased to 8, the neighborhood information becomes less, and accuracy also decreases (96.6%). When *k* increases, the neighborhood field is enlarged, the model captures more spatial information, and the accuracy can reach 97.19%. The number of points far from the center increases, and those points have less correlation with the center point, so the mean accuracy improves slowly. As *k* increases, the number of convolution operations in the neighborhood of each point increases, so the prediction speed becomes slower.

## 5. Conclusions

This paper presents a method to identify some components of the catenary in point clouds based on deep learning. Experiment results show that the proposed method has an excellent performance. In our method, based on the classification results of the single frame identify model, it can segment the components in different scenes with different models. The model which processes point independently can obtain a segmentation of the wire and simple dropper environment with good performance, and the proposed model which contains neighborhood information is more suitable for the complex scenes.

Based on the recognition results and the 3D coordinate information, it can measure the geometric parameters of OCS components, such as the height and stagger of the contact wire, the slope of the steady arm, the mast gauge (the distance from the center of the railway line to the inside edge of the pole). The point clouds in the areas where the components are connected are hard to be classified very accurately, so some are misidentified. Besides, misidentification outliers may occur, although with high accuracy. These effects on parameter measurement can be reduced by point cloud filtering or clustering.

In the future, we plan to integrate the single frame classification model and segmentation model. The measurement train is a common catenary inspection equipment, and we will test the performance of the proposed method on the data collected by a moving measurement train.

## Figures and Tables

**Table 1:** Segmentation accuracy (%) comparison.

Method	Mean	CatenaryWire	ContactWire	Pole	Insulator	Dropper	Cantilever	RegistrationArm	SteadyArm
PointNet	94.52	98.99	99.14	99.73	96.03	89.67	87.03	93.59	92.02
Ours (without local features)	95.55	98.97	99.52	**99.85**	96.57	91.61	92.06	93.6	92.19
Ours (k = 16)	**97.01**	**99.45**	**99.79**	99.8	**97.36**	**96.39**	**92.81**	**95.23**	**95.27**

**Table 2:** Comparison of the average inference time per sample.

Method	Inference Time
PointNet	0.0014 s
Ours (without local features)	**0.0012 s**
Ours (k = 16)	0.0031 s

**Table 3:** Comparison of our model with different numbers of nearest neighbors.

Number of Nearest Neighbors (k)	Mean Accuracy (%)	Inference Time (s)
8	96.6	**0.0030**
16	97.01	0.0031
32	97.1	0.0034
64	**97.19**	0.0041
